# Forecasting SARS-CoV-2 spike protein evolution from small data by deep learning and regression

**DOI:** 10.3389/fsysb.2024.1284668

**Published:** 2024-04-09

**Authors:** Samuel King, Xinyi E. Chen, Sarah W. S. Ng, Kimia Rostin, Samuel V. Hahn, Tylo Roberts, Janella C. Schwab, Parneet Sekhon, Madina Kagieva, Taylor Reilly, Ruo Chen Qi, Paarsa Salman, Ryan J. Hong, Eric J. Ma, Steven J. Hallam

**Affiliations:** ^1^ International Genetically Engineered Machine (iGEM) Team, University of British Columbia, Vancouver, BC, Canada; ^2^ Department of Botany, University of British Columbia, Vancouver, BC, Canada; ^3^ Department of Zoology, University of British Columbia, Vancouver, BC, Canada; ^4^ Department of Microbiology and Immunology, University of British Columbia, Vancouver, BC, Canada; ^5^ Department of Computer Science, University of British Columbia, Vancouver, BC, Canada; ^6^ Department of Chemical and Biological Engineering, University of British Columbia, Vancouver, BC, Canada; ^7^ Faculty of Land and Food Systems, University of British Columbia, Vancouver, BC, Canada; ^8^ Department of Cellular, Anatomical, and Physiological Sciences, University of British Columbia, Vancouver, BC, Canada; ^9^ Independent Researcher, Cambridge, MA, United States; ^10^ Graduate Program in Bioinformatics, University of British Columbia, Vancouver, BC, Canada; ^11^ Genome Science and Technology Program, University of British Columbia, Vancouver, BC, Canada; ^12^ Life Sciences Institute, University of British Columbia, Vancouver, BC, Canada; ^13^ ECOSCOPE Training Program, University of British Columbia, Vancouver, BC, Canada

**Keywords:** deep learning, regression, protein evolution, SARS-CoV-2, spike protein, small data, predictive model

## Abstract

The emergence of SARS-CoV-2 variants during the COVID-19 pandemic caused frequent global outbreaks that confounded public health efforts across many jurisdictions, highlighting the need for better understanding and prediction of viral evolution. Predictive models have been shown to support disease prevention efforts, such as with the seasonal influenza vaccine, but they require abundant data. For emerging viruses of concern, such models should ideally function with relatively sparse data typically encountered at the early stages of a viral outbreak. Conventional discrete approaches have proven difficult to develop due to the spurious and reversible nature of amino acid mutations and the overwhelming number of possible protein sequences adding computational complexity. We hypothesized that these challenges could be addressed by encoding discrete protein sequences into continuous numbers, effectively reducing the data size while enhancing the resolution of evolutionarily relevant differences. To this end, we developed a viral protein evolution prediction model (VPRE), which reduces amino acid sequences into continuous numbers by using an artificial neural network called a variational autoencoder (VAE) and models their most statistically likely evolutionary trajectories over time using Gaussian process (GP) regression. To demonstrate VPRE, we used a small amount of early SARS-CoV-2 spike protein sequences. We show that the VAE can be trained on a synthetic dataset based on this data. To recapitulate evolution along a phylogenetic path, we used only 104 spike protein sequences and trained the GP regression with the numerical variables to project evolution up to 5 months into the future. Our predictions contained novel variants and the most frequent prediction mapped primarily to a sequence that differed by only a single amino acid from the most reported spike protein within the prediction timeframe. Novel variants in the spike receptor binding domain (RBD) were capable of binding human angiotensin-converting enzyme 2 (ACE2) *in silico*, with comparable or better binding than previously resolved RBD-ACE2 complexes. Together, these results indicate the utility and tractability of combining deep learning and regression to model viral protein evolution with relatively sparse datasets, toward developing more effective medical interventions.

## Introduction

Viruses are responsible for millions of deaths and at least a third of all known infectious disease mortalities annually (Lozano et al., 2012; Marston et al., 2014; World Health Organization, 2022). The ability of a virus to infect its host is dependent on a “lock-and-key” mechanism, whereby glycoproteins decorating the viral surface interact with receptors on the host cell to induce a fusion event that results in entry of the viral particle (Choppin and Scheid, 1980; Davey et al., 2011). The specificity of the viral surface glycoprotein to a host cell receptor is known as its tropism and is considered one of the most crucial factors for new and persisting viral diseases (Majumdar and Jana, 2023).

Surface glycoproteins undergo strong selective pressure to maintain or shift their tropism, often leading to increased infectivity rates (Baranowski et al., 2001; Duffy et al., 2008). For example, the SARS-CoV-2 spike protein enables entry into human cells through binding of the angiotensin-converting enzyme 2 (ACE2) receptor (Kim et al., 2020; Ou et al., 2020; Zhang et al., 2020; Oudit et al., 2023). Over the course of the COVID-19 pandemic, thousands of variants have evolved (Koyama et al., 2020), and several were responsible for devastating outbreaks, such as the Omicron variant, which alone has 15 novel mutations in its spike protein receptor binding domain (RBD) that increased its virulence (Lupala et al., 2022; Oudit et al., 2023). The importance of the spike protein for host cell entry made it a logical target candidate for vaccines (Le et al., 2020). Therefore, it is evident that understanding the evolution of viral surface glycoproteins is imperative for establishing effective and preventative medical interventions for viral diseases. Moreover, the rate at which medicines can be produced is vital to saving lives and safeguarding immunity (Gao et al., 2023).

Predicting viral protein evolution has long stood as a grand challenge in biology. Previous efforts have typically been carried out using computational models that aimed to simulate general mechanisms of natural evolution, such as genetic drift (McCall, 2005; Huddleston et al., 2020). For example, the arrival of new influenza strains each year has encouraged the development of various models (e.g., Steinbrück et al., 2014; Łuksza and Lässig, 2014), which are useful in the development of annual flu vaccines (Morris et al., 2018). However, these models are ineffective at dealing with the highly dimensional factors of viral evolution and also have difficulty with incorporating uncertainty and stochasticity (Petrova and Russell, 2018; Perofsky and Nelson, 2020). Newer methods, particularly those involving machine learning, tend to extrapolate patterns from recorded evolutionary events, rather than impose theorized patterns of natural processes (Lee et al., 2016; Hie et al., 2021). Many of these methods implement deep learning, which is a subfield of machine learning that uses neural networks to learn from large amounts of data (Eraslan et al., 2019; Li et al., 2019). Deep learning has already been used to model viral evolution in a discrete context (Crossman, 2020; Sawmya et al., 2020; Younis, 2021; Han et al., 2023). However, such models are typically trained on vast amounts of data that are not available during early stages of a viral outbreak and tend to be impeded by the overwhelming dimensionality of protein sequence space (Pocrnic et al., 2016). Protein sequence space explodes combinatorially with each predicted amino acid, and there is no apparent forward notion of change from one amino acid to the next, further highlighting the importance of reducing the dimensionality issue in viral evolution.

To tackle the issues of data scarcity and dimensionality, neural networks called variational autoencoders (VAEs) have been implemented to reduce multidimensional data down to numerical values that still capture biologically relevant features (Pocrnic et al., 2016). VAEs can compress protein sequences into continuous latent space, which is a continuum of numerical values where similar values are located closely together. By learning to represent protein sequences as continuous numerical coordinates, VAEs have been used to capture biological information such as sequence mutations, novel cancer biomarkers, and protein family fitness landscapes (Riesselman et al., 2018; Ding et al., 2019; Simidjievski et al., 2019). Reducing protein sequences into lower-dimensional numerical encodings allows them to be further analyzed for evolutionary patterns that are otherwise difficult to detect, and substantially reduces the data size (Way and Greene, 2018). However, VAEs have not yet been integrated in a model for predicting protein evolution.

To find the trajectory of protein sequence evolution over time, Gaussian process (GP) regressions are highly effective as probabilistic models and have been widely implemented (Rasmussen and Williams, 2008; Das et al., 2018). GPs are a Bayesian learning technique that construct probability models of previously observed data, which they can make inferences from (Rasmussen and Williams, 2008). GPs have been used to model various protein properties (e.g., ligand-binding affinity or enzyme activity) with high accuracy (Romero et al., 2013). Regression modeling and time-series forecasting are common applications of GPs. For time-series regression, a GP can fit functions to a given set of data and time points and generate regression functions with associated probability distributions that allow for modeling of temporal trends (Cheng et al., 2019). GPs present an advantage over discrete models because their predictions are continuous, providing more granularity (Romero et al., 2013). The ability of GPs to quantify uncertainty helps to determine the validity of the outputs and utilizing them to model temporal trends also makes fewer assumptions on the shape of data distribution (Roberts et al., 2013; Romero et al., 2013), thus providing a more reliable model. Previous studies have shown the utility of GPs and/or latent spaces to study phylogenetic relationships, model protein stability, design proteins, and in inferring chemical species involved in biochemical interaction networks (Gao et al., 2008; Jones and Moriarty, 2013; Greener et al., 2018; Riesselman et al., 2018; Ding et al., 2019). However, the use of GPs and latent spaces for predicting unseen evolution on labeled timelines has not been employed.

**FIGURE 1:**
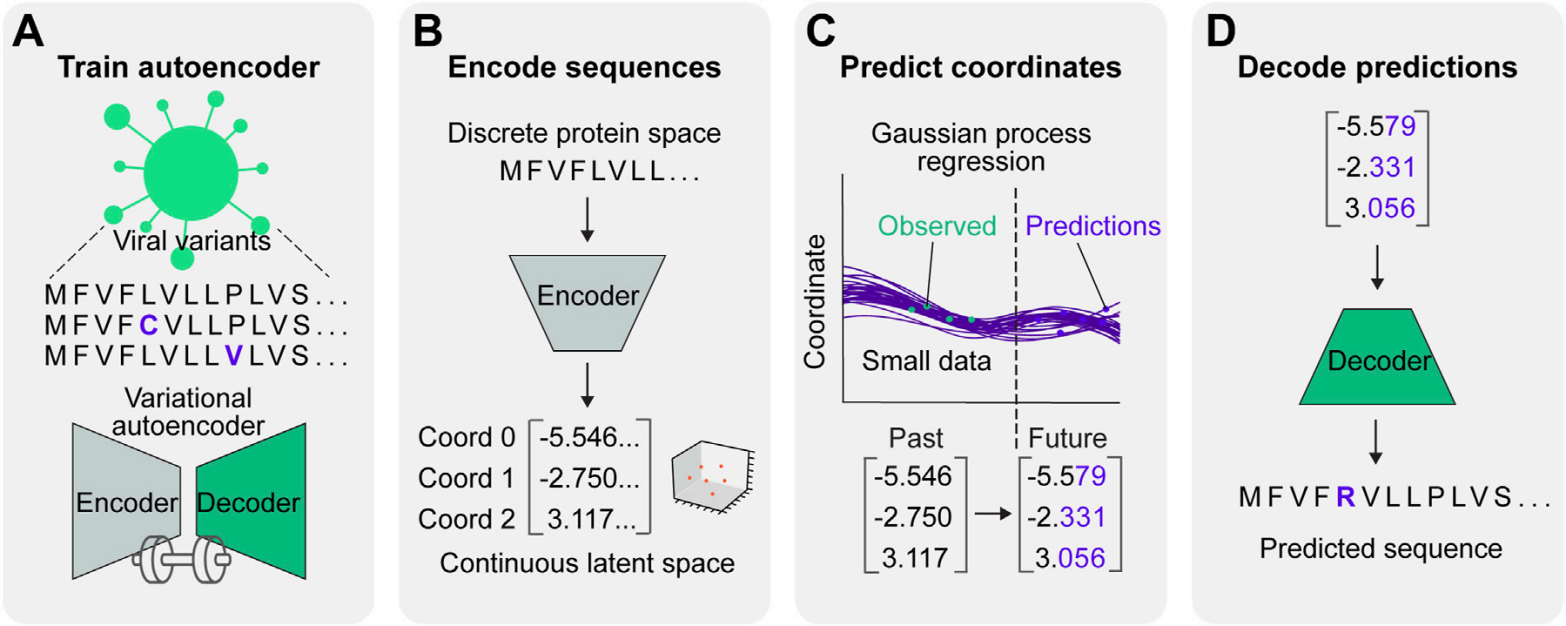
A predictive model for viral protein evolution. **(A)** Step 1: Profile and diversity analysis of SARS-CoV-2 spike protein sequences for neural network training. The diversity profile is used to generate a synthetic dataset of 20,000 spike protein sequences to train the variational autoencoder. **(B)** Step 2: Each sequence in the dataset is encoded into three continuous numerical variables (*i.e.*, coordinates) in continuous latent space. **(C)** Step 3: The Gaussian process regression charts the best statistical fit of the coordinates over time, and projects future coordinates in a given prediction timeframe. **(D)** Step 4: Projected coordinates are decoded into putative sequences resulting from SARS-CoV-2 evolution.

Here, we integrate a VAE and GP regression to create a synergistic framework for viral protein evolution prediction (VPRE) that requires a relatively small amount of input data to function (Figure 1). VPRE models protein evolution as a continuum of numerical coordinates, rather than as a discrete timeline of amino acid sequences. After compressing viral proteins with the VAE to create a biologically relevant latent space (Figures 1A, B), the GP projects the most statistically likely chronological trajectories of protein evolution (Figures 1C, D). To demonstrate VPRE, we used a small amount of early pandemic SARS-CoV-2 spike protein sequence data. We found that we could robustly train the VAE on a synthetic spike protein dataset generated from an early collection of real-time variants. As a proof-of-concept, we made predictions one, two, and five months into the future using only 104 sequences from Australia. VPRE predicted 17 variants, six of which were putative spike proteins that closely resemble the composition of spike proteins that appeared in real time, differing by only zero to three amino acids depending on the sequence. The most frequent prediction five months into the future was only one amino acid different from the most frequent spike protein in real world data. VPRE was also able to output novel variants it had not seen in the dataset. Novel variants in the receptor binding domain (RBD) were capable of binding human ACE2 *in silico* with docking scores similar to or greater than previously resolved crystal structures. Together, these results indicate the utility and tractability of combining deep learning and regression to model viral protein evolution with relatively sparse datasets.

## Results

### Capturing spike protein variation and training the variational autoencoder

**FIGURE 2:**
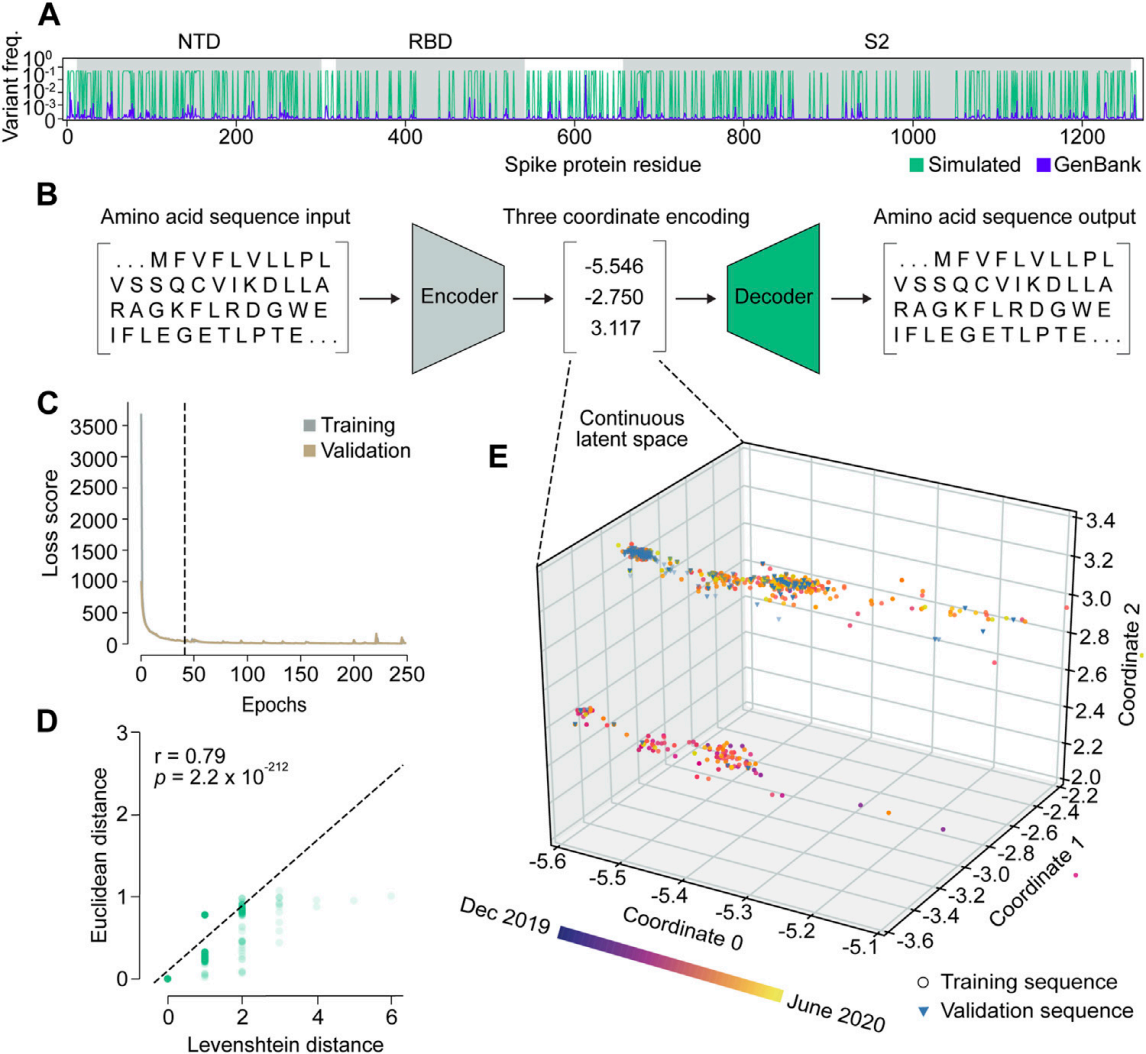
Variational autoencoder architecture and training. **(A)** Variant frequency at each amino acid position on spike proteins in the NCBI GenBank dataset (purple) and the simulated dataset (green). NTD, N-terminal domain; RBD, receptor binding domain; S2, S2 subunit. **(B)** Illustration of the variational autoencoder (VAE) architecture. Three latent dimensions, or coordinates, were set for VAE-translated variables. **(C)** VAE training loss curves. The dashed line indicates the number of epochs used in training the final model. **(D)** Correlation of Levenshtein distances of each sequence pair in the NCBI dataset and Euclidean distances of the corresponding latent representations from the VAE. The black dotted line is the fitted line. The significance threshold was adjusted by Bonferroni correction. *n* = 7,620. **(E)** Overview of latent representations of the viral spike protein sequences. Sequences collected prior to 30 May 2020, are grouped as a training dataset, and are represented by circles (*n* = 7,620). Sequences collected after 30 May 2020, are grouped into a validation dataset, and are represented by triangles (*n* = 1,914).

We set out to build VPRE from an early pandemic dataset to demonstrate its utility at that stage. The World Health Organization declared the COVID-19 viral disease a pandemic in March 2020. Our dataset included 9534 SARS-CoV-2 spike protein sequences collected internationally during the early months of the pandemic, between 25 January and 30 June 2020 (Supplementary Figure S1), or approximately 2 months before the pandemic was declared and approximately 3 months after. An initial obstacle when training the VAE was that the limited diversity in our relatively small dataset made it difficult for the neural network to identify patterns due to class imbalance (Supplementary Figures S1A, B). Across all sequences, the average mutation frequency per amino acid variant was 1.5 × 10^−4^ (Supplementary Figures S1C, D). As expected, we found that the spike protein is quite conserved, especially in its RBD, where mean variant frequency per amino acid was 6 × 10^−5^ (Supplementary Figure S1C). To improve the VAE training, we simulated 20,000 spike proteins with an amplified but equal chance of mutation at any of the mutation sites seen in the original protein sequences, while the conserved regions were maintained (Figure 2A). Consequently, most variant frequencies at each position rose to approximately 0.3 or 0.5, given that there were many amino acid positions with two or three variants. This approach increased the VAE’s ability to encode and decode rare variants in the dataset, which ensured that low-frequency variants were represented in the GP input and could be decoded accurately if predicted (Figures 2B–E).

We trained the VAE for 41 epochs, determined by an early stopping function, where a single epoch included one round of encoding and decoding. This was followed by the calculation of a difference score between the input and output sequences, which represented the loss or error of the model (Figure 2C; Supplementary Figure S2). As a simple means to verify whether the sequence encodings output by the VAE accurately captured differences in the spike protein sequences, we compared the Euclidean distances between the numerical latent coordinates to the Levenshtein distances between the amino acid sequences (Figure 2D). A Euclidean distance is the length of a line segment between two data points in geometric space, while a Levenshtein distance is a metric for measuring the number of differences between two strings (Levenshtein, 1966). The two variables were strongly correlated (*r* = 0.79), suggesting that sequences became increasingly different as their distance in latent space increased, and that variation within amino acid sequences was well-captured in the VAE-encoded numerical coordinates.

### Encoding spike proteins in continuous latent space using the variational autoencoder

After training the VAE on 20,000 simulated sequences, we encoded 7,620 spike protein sequences collected before 30 May 2020, into three latent dimensions, or numerical coordinates (Figures 2B, E). The continuous latent space representation created by our sequence encodings separated into two major populations: one proximal to the sequences collected near December 2019, and the other proximal to those from May 2020 (Figure 2E). We used the remaining 1914 sequences from the dataset collected after 30 May 2020, to validate the latent space generated by the first sequence encodings. As expected, the validation data appeared in the latent space within the population of sequences proximal to May 2020. This suggests that the VAE could learn a latent variable model and the parameters of the probability distribution modeling the input data.

### Predicting evolutionary trajectories of spike proteins using Gaussian process regression

To forecast novel predictions, we input each coordinate of the encoded sequences from the VAE into individual GPs. Each GP performed a regression analysis on the VAE coordinates to find the best fitting functions to the data points in chronological order. After this training period, the functions were projected into the future to predict the sequences of the most statistically likely spike proteins that might evolve based on previous evolutionary patterns. Because GP predictions are continuous coordinates and amino acid sequences are discrete, multiple coordinate triplets can represent the same amino acid sequence. As a result, a frequency index can be calculated for each predicted sequence, which we used to estimate their likelihood.

**FIGURE 3:**
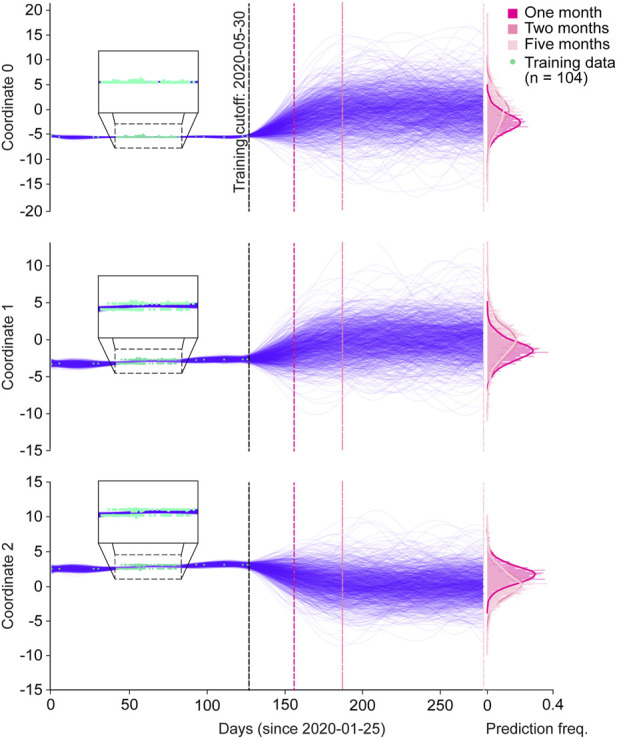
Spike protein evolution projected up to five months into the future by the Gaussian process. Trajectories of latent representations of sequences analyzed by the Gaussian process (GP) for each encoded coordinate (purple lines; *n* = 1,000 predictions), with the training coordinates overlaid on the training period (green dots; *n* = 104). After training the GP on sequences up until 30 May 2020, predictions were made one month (dark pink dotted line), two months (semi-dark pink dotted line), and five months (light-pink dotted line) into the future. The corresponding frequency distributions of each prediction period are shown on the right.

As a proof-of-concept, we tested the ability of GPs to predict spike protein evolution on a very small dataset, by training on sequences from Australia collected prior to 30 May 2020 (*n* = 104), and projecting the trajectories of 1,000 sequences one, two, and Five months into the future (Figure 3; Supplementary Figure S3A). We chose Australia with the assumption that it would allow us to simulate an isolated and simplified phylogenetic pathway for SARS-_CoV-2 spike proteins, under the hypothesis that Australia as an island continent was more isolated and therefore, less subject to external factors contributing to variant emergence. Moreover, within the entire dataset Australia had the most sufficient spike protein data available for our analysis when compared to other island nations. Within the training period, the functions of all three coordinates tightly fitted with the training sequences (Figure 3 magnified boxes). In the first two months of predictions, the range of coordinate values expanded, but then stabilized throughout the five month prediction period. This can also be seen in the frequency distributions for each coordinate and their respective prediction periods, where the predicted values for each coordinate generally followed a normal distribution, and months two and five appeared to have similar value distributions. Clear clustering of the training data points is seen in coordinates 1 and 2, suggesting the presence of two dominant spike protein sequences within the Australian dataset.

### Variant predictions closely represent real-time evolution

**FIGURE 4:**
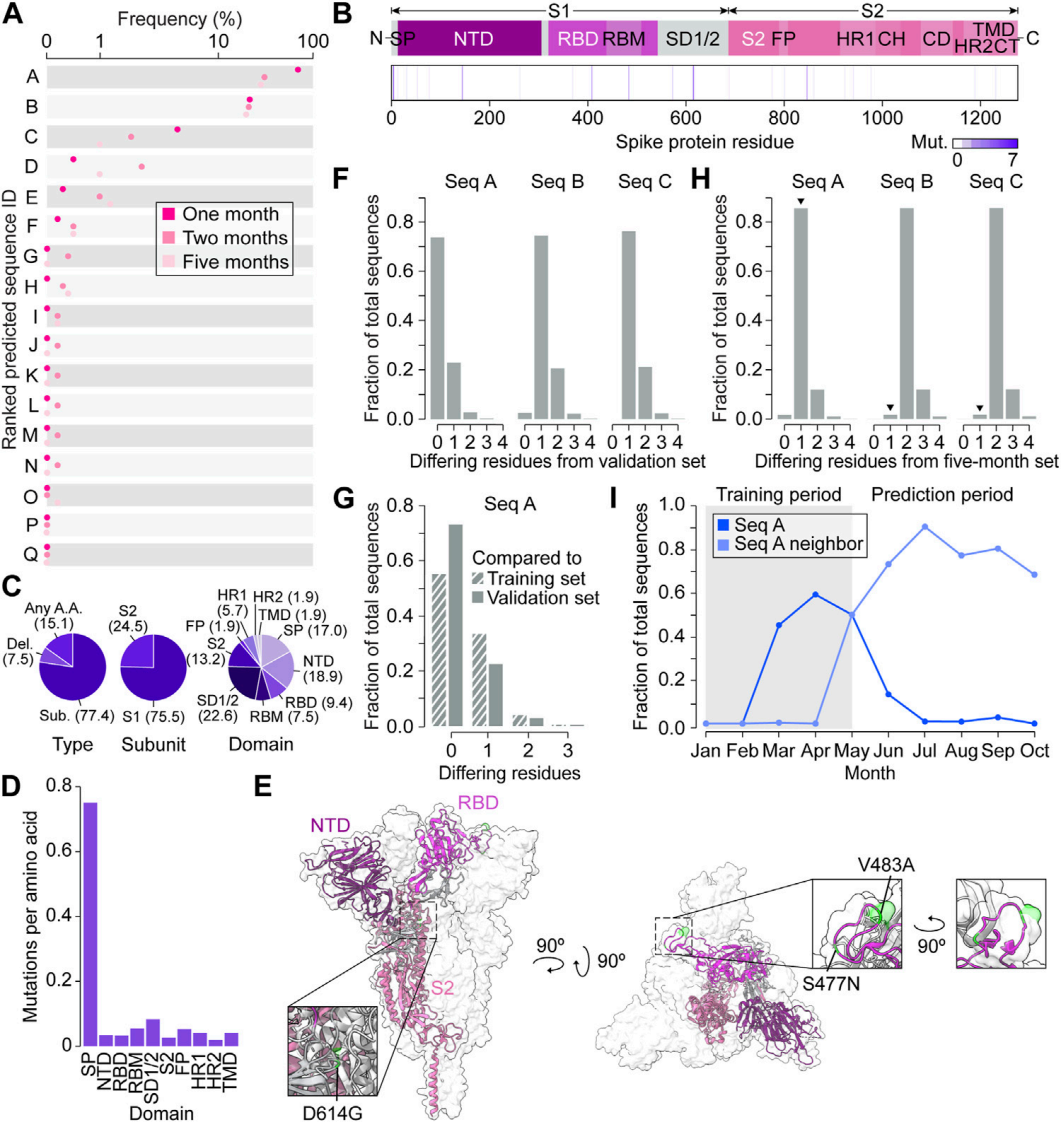
Spike protein variant predictions closely represent real-time evolution in the prediction timeframe. **(A)** Predicted amino acid sequences decoded from three Gaussian process (GP) regressions performed on coordinates 0, 1, and 2. The frequency of predictions is ordered from highest to lowest from A to Q. **(B)** Frequencies of predicted mutations in the spike protein. CH, central helix; CD, connector domain; CT, cytoplasmic tail; FP, fusion peptide; HR1, heptad repeat region 1; HR2, heptad repeat region 2; NTD, N-terminal domain; RBD, receptor binding domain; RBM, receptor binding motif; S1, S1 subunit; S2, S2 subunit; SD1/2, subdomain 1 and subdomain 2; SP, signal peptide, TMD, transmembrane domain. **(C)** Percentages (in brackets) of predicted variants categorized by type of mutation, spike protein subunit, and spike protein domain. **(D)** Predicted mutations per amino acid of each spike protein domain. **(E)** The top three variant predictions mapped onto the crystal structure of a trimeric prefusion spike protein ectodomain. Only one of the three protomers is highlighted. PDB: 6XR8 (Cai et al., 2020). **(F)** Number of amino acid differences between the top three predictions and validation sequences collected in Australia up to 1 month after 30 May 2020 (*n* = 81). **(G)** Number of amino acid differences between the most frequent prediction (Seq A) and the GP training sequences (*n* = 104) and validation sequences (*n* = 81). **(H)** Number of amino acid differences between the three most frequent GP predictions and Australian sequences collected within the five month prediction period (between May 30 and 30 November 2020). Arrowheads indicate the sequences’ nearest neighbors. **(I)** Monthly frequency of Seq A and its nearest neighbor across the entire Australian spike protein dataset.

The VAE decoded the 1,000 coordinate triplets predicted by the GP at the end of five months into 17 amino acid sequences (Figure 4A). Following a seed and extend BLAST search, we found that the top two predictions were existing spike proteins and the other 15 were novel sequences. All 17 predictions contained a total of 53 mutations, with each appearing between one and seven times depending on the position (Figure 4B). The majority of mutations were substitutions (77.4%) and occurred in the S1 subunit (75.5%) (Figure 4C). By calculating the frequency of mutations proportional to spike domain size, we could measure whether each domain appeared to have a similar mutation rate per amino acid (Figure 4D). Interestingly, we found that the mutation rate varied between domains, and the signal peptide had a particularly high mutation rate compared to others, further indicating that biologically relevant information was captured in VPRE’s predictions. We summarized the changes in the predicted proteins compared to the original strain from Wuhan (QHU36824) in Supplementary Table S1.

We next focused on characterizing the top three predictions, Seq A, B, and C, with Seq A being the top prediction followed by Seq B and C (Supplementary Table S2). Compared to the last sequence at the training cutoff date of 30 May 2020 (hereafter referred to as “Seq 053020”), all top three predictions contained variants S477N. Seq A and C contained D614G, and Seq C contained additional variants L5F, V483A, and A845D (Supplementary Table S3). The three most frequent mutations across all three sequences were S477N, D614G, and V483A, which appear in the receptor binding motif (RBM), subdomain 1 and 2 (SD1/2), and RBM, respectively (Figures 4B, E). It was reported in previous studies that S477N can change antibody accessibility (Harvey et al., 2021) and D614G increases infectivity (Korber et al., 2020). While Seq C had novel variants L5F and A845D that did not have much functional characterization in deep mutational scanning, V483A is likely to impact antigenicity (Harvey et al., 2021).

To benchmark the functionality of VPRE, we compared the top three predictions to the sequences collected in Australia one month after the training data cutoff date of 30 May 2020 (*n* = 81, Figure 4F; Supplementary Figure S3A). The top prediction comprised over 70% of the validation dataset, while the second- and third-most probable predictions were identical to less than 25% and 5% of the validation sequences, respectively. When comparing the top prediction to our GP training sequences (*n* = 104), around 55% were identical to our top prediction (Figure 4G). These data suggest that our GP worked as expected even when trained on a small dataset, given that it predicted the most dominant sequence it was trained on, which was also the most dominant sequence present in Australia during the prediction period. The top two predictions being identical to existing spike proteins in Australia also suggests that the VAE could reproduce accurate spike protein sequences.

To compare the three most frequent predictions against more data and evolutionary time, we retrieved 8,407 additional Australian spike protein sequences collected between the training cut-off date of 30 May 2020, and 30 November 2020 (the five month prediction period) (Figure 4H; Supplementary Figure S3B). Over 85% of the newly retrieved sequences differed from our most frequent prediction by only one amino acid (N477S), and over 90% of these nearest neighbors were identical to each other. The most common nearest neighbors of the second- and third-most frequent predictions differed by only two amino acids from the predictions. When tracing the frequency patterns of the top prediction and its nearest neighbor in our Australian dataset between January and November 2020, we found that the top prediction was the most prevalent spike protein up until May (Figure 4I). After May, the top prediction’s nearest neighbor became the most prevalent spike protein in Australia, emerging in April, reaching a frequency of around 90% by July and outcompeting our predicted sequence. Taken together, the GP’s top prediction was off by only a single amino acid when extrapolating the most dominant spike protein five months into the future.

### Novel variants produced by VPRE bind ACE2 *in silico*

**FIGURE 5:**
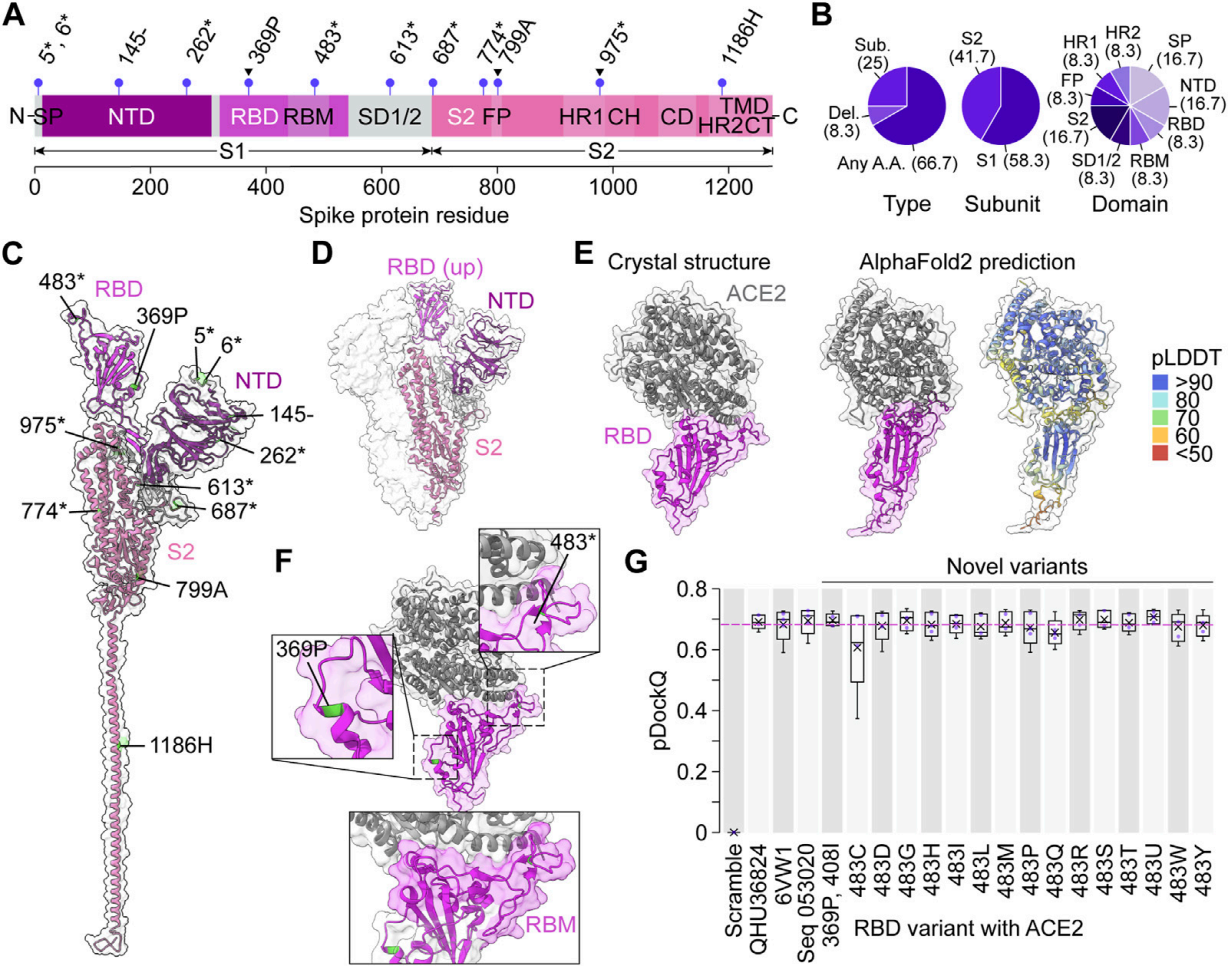
Novel spike protein variants bind ACE2 *in silico*. **(A)** Unique variant positions produced by the variational autoencoder that were unseen in the training dataset, indicated by purple lollipops. Arrowheads indicate conserved positions in the training dataset. Asterisks represent any amino acid, dashes represent deletions. CH, central helix; CD, connector domain; CT, cytoplasmic tail; FP, fusion peptide; HR1, heptad repeat region 1; HR2, heptad repeat region 2; NTD, N-terminal domain; RBD, receptor binding domain; RBM, receptor binding motif; S1, S1 subunit; S2, S2 subunit; SD1/2, subdomain 1 and subdomain 2; SP, signal peptide, TMD, transmembrane domain. **(B)** Percentages (in brackets) of novel predicted variants categorized by type of mutation, spike protein subunit, and spike protein domain. **(C)** The novel variant predictions mapped onto the crystal structure of a single prefusion spike protein protomer in the “up” conformation. PDB: 6VYB (Walls et al., 2020). **(D)** Crystal structure of a trimeric prefusion spike protein with a single RBD in the “up” conformation. PDB: 6VSB (Wrapp et al., 2020). **(E)** Crystal structure of a spike RBD binding human ACE2 compared to the AlphaFold2 prediction of the same sequence. PDB: 6VW1 (Shang et al., 2020). **(F)** The two novel variants in the RBD mapped on the crystal structure in **(E)**, with the RBM highlighted below. **(G)** pDockQ scores of AlphaFold2-predicted RBD-ACE2 binding models (*n* = 5 models each). In each box, x = mean, middle line = median, lower hinge = 25th percentile of the data, upper hinge = 75th percentile of the data, whiskers = 1.5 * interquartile range. Pink dotted line represents the mean pDockQ score of 6VW1.

To investigate whether the model produced amino acid variants that were not seen in the training dataset, we compared the 17 predicted sequences against all training sequences (Figure 5A; Supplementary Table S4). We found 11 novel amino acid substitutions and four deletions across all spike protein domains except for the furin cleavage sequence, central helix, connector domain, transmembrane domain, and cytoplasmic tail (Figures 5B, C). Interestingly, there were three predicted amino acid variants at conserved regions within the training data (Figure 5A).

To understand the functional significance of the predicted variants, we turned to published deep mutational scanning studies. Deletion of amino acid at position 145 is present in Seq E, G, H, and P, and is found to abolish neutralizing antibody 4A8 binding (Figures 5A, C; McCarthy et al., 2021). Moreover, there are eight positions predicted to mutate to any amino acid. Two of them, position 483 (in Seq L) and position 687 (in Seq F), are likely to impact antigenicity (Harvey et al., 2021) and can lead to possible antibody-escape (Dadonaite et al., 2023), respectively. These results not only indicate that the model was able to produce sequences other than those that it was trained on, but it also shows these novel predictions are biologically meaningful.

Next, we sought to measure the fitness of variants produced by VPRE by testing their binding ability to human ACE2. The spike protein RBD interacts with ACE2 when exposed in the “up” position (Figure 5D; Yan et al., 2021). For this reason, we decided to model RBD-ACE2 complexes. Two sequences, Seq P and Seq L contained novel variants in their RBD, which were 369P and 408I (Seq P), and 483* (Seq L), where “*” represents any amino acid. Given that VPRE considered any amino acid possible at position 483, we extracted only the novel amino acids at that position (see Supplementary Table S5). In total, we could model 16 novel predicted variants within the spike protein RBD and perform a binding analysis with ACE2. The binding analysis consisted of modeling the multimeric structure of the RBD-ACE2 complex using AlphaFold2-multimer (Evans et al., 2021) and calculating a predicted DockQ (pDockQ) score based on the contacts found in the structure (see Methods for details; Bryant et al., 2022; Lim et al., 2023). DockQ is a widely used protein-protein docking quality metric on a scale of 0–1, where acceptable models give a score greater than 0.23 (Basu and Wallner, 2016). Scores above ∼0.5 are considered medium quality, and above ∼0.8 are considered high quality. More recently, DockQ scoring has been adapted to measure the accuracy of AlphaFold2-predicted multimers (Bryant et al., 2022). Using the pDockQ scoring system, we compared ACE2 binding of the 16 novel RBD variants with AlphaFold2 models of a randomly scrambled RBD sequence (Scramble), the Wuhan reference sequence QHU36824, a previously resolved crystal structure of the RBD-ACE2 complex (6VW1, Shang et al., 2020), and Seq 053020 (Figures 5E–G; Supplementary Figure S4). As expected, the Scramble complex scored 0 on average, due to AlphaFold2 being unable to fold any coherent structure from the sequence (Supplementary Figures S5, S6). All other structures had mean pDockQ scores between ∼0.6 and 0.7, with nine out of 16 novel variants having mean scores greater than 6VW1. In general, all complexes scored similarly, with the exceptions of Scramble and 483C. Together, these data suggest that novel variants produced by VPRE are likely fit mutations capable of binding ACE2.

## Discussion

A major bottleneck in modeling and predicting viral evolution in real time is the amount of data available, especially in the early stages of a pandemic when sequencing data is relatively sparse. This was evident in the COVID-19 pandemic, which prompted us to build a predictive model with the aim of extracting as much information as possible from limited data. VPRE integrates a VAE and GP regression to predict the mutational trajectory of viral proteins with a small amount of data. The VAE encodes protein sequences as continuous numbers, and the GP regression fits the latent numerical representations over time, which allows the model to continue charting changes in variants into the future. Finally, the VAE decodes the predicted numbers back to protein sequences. With this approach, we analyzed the first seven months of available SARS-CoV-2 sequences and showed that VPRE can learn from limited data and make biologically meaningful predictions.

We applied VPRE to model variants in Australia from December 2019 to May 2020 and predict variants that will be present in June, July, and October 2020, or one month, two months, and five months into the future, respectively. We first focused on the top three predictions in our five month prediction window, Seq A, B, C (naming follows the order of likelihood of occurring). Compared to the last protein sequence in May 2020, all top three predictions contained S477N. Seq A and C contained D614G, and Seq C contained additional variants L5F, V483A, and A845D. The variants S477N and D614 were seen in the data that was collected during and one month after the training period. Although they were not novel variants, this finding suggests that no variants rose to dominance within a one month timeframe, corresponding with the evolutionary trends we observed in Australia over this period. Several other studies have examined the functional differences of numerous spike protein variants. For substitutions at position 477, structural studies suggest a change in antibody accessibility, and S477N could lead to escape of mAb neutralization (Harvey et al., 2021), and the substitution S477I is among the forecasted variants that REGN10933 (casirivimab) targets (Maher et al., 2022). In addition, our model predicted a variant V483A. Interestingly, the variant V483I circulated in the UK (Harvey et al., 2021), and the variant V483F is forecasted and targeted by LY-CoV555 (bamlanivimab) (Maher et al., 2022), suggesting position 483 may be important for antigenicity. Moreover, there are variants with low frequencies in our predictions, at positions 368, 798, and 1,185, where no mutations were seen in the training dataset. Although structural and mutational screening studies thus far have not indicated these positions as important for viral fitness, their presence in our predictions still suggests that VPRE is not overfitted to the training dataset and shows that VPRE can make predictions beyond what it has seen before. Overall, VPRE’s performance is sensible in both biological and computational perspectives and is promising given the small amount of training data.

A main advantage of VPRE is that it models evolution in a continuous space rather than with discrete protein sequences. Predicting mutational trajectories with VPRE is essentially a two-step process where the VAE encodes sequences and the GP models evolution. In both steps, there are advantages in using continuous numbers. First, our results suggest that the VAE was able to capture the complexity of proteins by reducing amino acid sequences of >1,000 characters (*i.e.*, dimensions) into only three continuous numerical values. This transformation from discrete amino acids to continuous numbers provides richer biological information for modeling. For instance, VPRE can account for different mutations at the same position by encoding them as different numbers, whereas traditional methods of calculating a Hamming or editing distance between two sequences do not distinguish the nature of the mutations (Pinheiro et al., 2012). Additionally, a mutation may impact more than one coordinate in our method and therefore we can also model its effect in a larger context. Second, mutations are spurious and reversible, which makes it difficult to perceive a forward notion of change, and as such they could be better modeled using a continuous approach. In the GP portion of the model, we saw that the numerical values vary greatly when we project the regression into the future, capturing the spurious nature of mutations. We also saw that despite the spread of the numerical values, they largely map back to the same sequence, indicating that some of the variability is not enough to lead to actual changes. This variability is intriguing and future efforts should investigate it further regarding viral fitness.

It should be noted that VPRE is prone to bias intrinsic to the training dataset, which could be challenging especially for data under a short time series. To increase our VAE’s ability to detect variants, we synthesized a dataset of 20,000 spike protein sequences with equally amplified mutation frequencies. Amplifying the mutational variation might have introduced unnatural features in spike proteins or biased the network’s ability to model variants better than conserved amino acids. We expect the performance of the model to improve with continual learning. As more data becomes available, the VAE can be continuously trained and thus improve on its ability to encode sequences. VPRE also had no direct measure of antigenic shift or fitness other than the extent of these characteristics captured in the spike protein sequences over time. By performing a docking analysis, we were able to interpret the fitness of VPRE’s predictions *in silico*, but further experiments are necessary to validate the function of the predicted variants, especially at the scale of the whole spike protein trimer.

In addition, VPRE can be easily adapted to model other proteins. For instance, a VAE and GP was used to infer the evolutionary landscape within protein families such as fibronectin type III domain, cytochrome P450, and staphylococcal nuclease (Ding et al., 2019). A VAE was also used on luciferase-like oxidoreductases to generate functional variants (Hawkins-Hooker et al., 2021). Further efforts are warranted to apply the VPRE framework to learn evolutionary information from other types of proteins. When there is data spanning a longer timeframe, we can apply the model on representative sequences of a week or longer to increase the robustness of the model as well as predict further into the future.

VPRE was designed to tackle the problem of *in silico* protein evolution using small data, with the added benefits of reducing the data size and potentially enhancing the amount of information that can be extracted from sequences. We envision that a computational model such as VPRE might be paired with *in vitro* efforts in laboratories and medical settings for the best prediction outcomes. For example, VPRE-predicted spike protein RBDs could be expressed in a yeast surface display system to assay ACE2 binding ability (Starr et al., 2020) or whole spike mutants could be displayed on mammalian cell surfaces (Javanmardi et al., 2021) or pseudoviruses (Nie et al., 2020; Dadonaite et al., 2023) and tested for antibody evasion using blood serum samples from vaccinated individuals. Overall, VPRE opens further investigation into evolutionary models that seek to improve epidemiological efforts and public health intervention systems, towards mitigating the harmful mutational dynamics of diseases.

## Materials and methods

### Data acquisition and construction

The VPRE training dataset consisted of 9534 SARS-CoV-2 spike protein sequences from around the world at varying time points throughout the pandemic (Supplementary File S1). These were downloaded along with their corresponding metadata from NCBI Genbank on 13 August 2020. The five month VPRE validation dataset from Australia consisted of 8,488 spike protein sequences, which were downloaded on 15 January 2021. Incomplete sequences containing gaps (dashes and asterisks) and sequences with ambiguous amino acids (null, B, Z, X) were removed to ensure high quality data.

A training set of 20,000 semi-random mutated spike protein sequences was generated algorithmically by ensuring equal representation in the dataset of any amino acid substitution that occurred at least one time in the spike protein dataset (Supplementary File S2). The algorithm started with the consensus sequence of our analyzed sequences, and went through each position stepwise, presenting with equal likelihood any point mutation observed in the data. This was repeated until 20,000 unique sequences had been created, with the effect of amplifying the presence of infrequent point mutations so that they could be more accurately decoded from predictions made by the GP. No change was made to the analyzed dataset, which remained as sequenced, and only underwent a multiple alignment prior to processing by the GP. The training dataset maintained all the same conserved regions as the real-world data.

### Encoding and decoding amino acid sequences with the variational autoencoder

As a preprocessing step, training set sequences were aligned by progressive alignment via the Multialign function in the MATLAB bioinformatic toolbox (Mathworks, 2021). The aligned sequences were padded with asterisk (*) characters to maximal length and one-hot encoded in order to yield binary matrix representations of the SARS-CoV-2 spike protein sequences that could be input into our deep learning model.

The VAE consisted of an encoder and decoder network, where the encoder compressed the sequence data (one-hot encoded amino acid sequences) to its latent embedding and the decoder decompressed the sequence data from its latent embedding. The VAE was implemented in Keras (version 2.4.0) (Chollet, 2015) using a TensorFlow backend (version 1.4.0) (Abadi et al., 2016). In the encoder, the number of latent dimensions was set to three to allow for easier visualization, and thus each sequence was compressed to three numerical coordinates. The latent space distribution was defined with a latent mean and logarithmic variance. Both were calculated with Keras Dense with one-hot encoded training and with an input dimension of three. A standard sampling layer and a Dense layer were created in the encoder. The sampling layer randomly sampled data from latent space following a normal distribution with a mean of zero and a standard deviation of one. The Dense layer mapped the sampled data points to the latent distribution. The decoder was constructed using the encoded data as input and the last layer of the autoencoder as output.

The VAE model was compiled with an Adam optimizer and custom-built loss function. The loss function was the sum of a reconstruction term and a regularization term (expressed as the Kullback-Leibler divergence between the distribution returned by the encoder and a standard normal distribution):

Loss=reconstruction loss+KL divergence regularization term,

$$Loss=BinaryCrossEntropy\left(x,\hat{x}\right)+\frac{-1}{2}\left(1+\log\sigma_{z}^{2}\;-\;\mu_{z}^{2}-\sigma_{z}^{2}\right),$$

where x represents the input data, $\hat{x}$ represents the reconstructed data, $\sigma_{z}^{2}$ represents the variance of the latent distribution, and $\mu_{z}$ represents the mean of the latent distribution. The reconstruction loss served as a measure of the efficacy of the encoder-decoder, as it represented the difference between the reconstructed (decoded) sequences and the input sequences (calculated using binary cross-entropy). Over training, the reconstruction loss was ultimately minimized. The regularization term helped in learning well-formed latent spaces and reducing overfitting during the training process (Phan et al., 2020). An early stopping function was also applied with a patience parameter of two in order to stop training once the validation loss metric had stopped improving for two consecutive epochs, thus avoiding overfitting.

### Modeling the trajectory of spike protein evolution with Gaussian process regressions

A GP was used to model temporal trajectories of each coordinate of Australian sequences encoded by the VAE. After removing duplicated sequences from each day to simplify the model, 185 sequences were obtained (Supplementary File S3). Data from sequences that were collected up to 31 May 2020 (*n* = 104) were used as a training dataset for the GP, and the rest from June 1 to June 30 (*n* = 81) were used for validation.

The PyMC3 package (version 3.11) (Salvatier et al., 2016) was used to construct the GP model. To model a temporal axis in the GP, an array was constructed to represent the number of days since the first sequence collection in Australia. The other axis in the GP consisted of the coordinate values from the VAE.

The GP was defined as $Y\;∼\;GP\left(K\left(x,x^{\prime }\right),µ\left(x\right)\right)$ , adapted from Eric Ma’s Flu Forecaster (https://github.com/ericmjl/flu-sequence-predictor/blob/master/flu-forecaster.ipynb) with a GP latent variable implementation sample (Salvatier et al., 2016).

First, the covariance function was defined as an exponentiated quadratic function. The exponentiated quadratic kernel is a popular kernel used in GP modeling, thus it was chosen as a starting point for modeling the data. Because the VAE coordinates were modeled individually, an input dimension of 1 was used for the exponentiated quadratic kernel. The GP model was computed as follows:

$$K\left(x,x^{\prime }\right)=e^{s\left(x\right)}\times \left(\frac{{\left(x-x^{\prime }\right)}^{2}}{{2l}^{2}}\right)$$

$$e^{s\left(x\right)}\;∼\mathrm{Uniform}\left(-10,5\right)$$

l ∼ Uniform 0,30

$$µ\left(x\right)\;∼\;0$$

The Uniform function in PyMC3 was used to construct the exponent and Theano was used to construct the exponentiation, followed by a deterministic transformation by using the Deterministic function from PyMC3 (Al-Rfou et al., 2016; Salvatier et al., 2016).

Second, a Student’s T log-likelihood distribution was defined to model uncertainties in the covariance function and the input data, adapted from a PyMC3 tutorial (https://docs.pymc.io/notebooks/GP-Latent.html):

df ∼ Gamma 2,1,where df=degrees of freedom

1lam ∼ HalfCauchy 0,5,where lam is a scale parameter

Lastly, the covariance and the mean function were assembled in a Latent GP model. The Exponentiated Quadratic covariance function and the time array were defined for a Latent GP. A Uniform log-likelihood distribution was applied to describe length-scale, as well as a HalfCauchy distribution and a Gamma distribution to define the uncertainty in the covariance function and to model the noise. The VAE coordinates were input as observed prior.

### Extrapolating the trajectory of Gaussian process models

To extrapolate the trajectories the GP predicted, a new time array was set from 0 to 120 + x, where 120 was the number of training days and x represented the number of days into the future to predict. The variable x was chosen as x = 30, 60, and 150 to predict one, two, and 5 months into the future. The new time array was applied on the GP and a conditional distribution of the predicted functions was obtained with the new input time values using the *conditional* function. 1,000 samples were drawn from the GP posterior for each of the three VAE coordinates and merged into 1,000 triplets to represent the predicted numerical representations of spike proteins. The triplets were decoded by the decoder of the VAE to obtain predicted spike proteins. The likelihood of each sequence existing in the predicted timeframe was estimated based on the fraction of the 1000 GP predictions that translated exactly to the sequence. Additional packages used in the pipeline include numpy (version 1.19.5) (Harris et al., 2020), SciPy (version 1.4.1) (Virtanen et al., 2020), and pandas (version 1.1.5) (McKinney, 2010).

### Modeling protein structures and *in silico* protein binding analysis

All protein structures and mutations were visualized using UCSF ChimeraX (version 1.5) (Pettersen et al., 2021). Labels were added to structures in Adobe Illustrator (version 26.5).

To analyze the fitness of novel mutants in VPRE-predicted spike proteins, we predicted the structures of spike RBDs in complex with human ACE2 using AlphaFold2-multimer (Evans et al., 2021; Jumper et al., 2021). AlphaFold2-multimer was run using Colabfold (version 1.5.2-patch) with High-RAM A100 GPUs (Mirdita et al., 2022). For every case, five models were run with three recycles each. The Colabfold pipeline generated all multiple sequence alignments (MSAs) and template inputs and used the paired and unpaired MSAs from MMseqs2 (Steinegger and Söding, 2017). To score protein interactions, we used a Python 3 Colabfold binding analysis script (Lim et al., 2023; https://zenodo.org/record/8223143) which finds residue contacts by iterating through each residue of a protein chain and determines its position and confidence relative to the residues in other chains. We set the threshold for a binding contact as a pair of residues that have an average predicted local distance difference threshold (pLDDT) score greater than 50, a minimum predicted alignment error (PAE) of less than 15 angstroms, and a maximum distance of two non-hydrogen atoms as eight angstroms. For each interaction, a predicted DockQ (pDockQ) value was calculated by the empirically derived formula (Bryant et al., 2022) made from the docking quality metric DockQ (Basu and Wallner, 2016):

$$pDockQ=\frac{0.724}{1+e^{-0.052\left(x-152.611\right)}}+0.018$$

$$\text{where }x=\mathrm{average interface pLDDT}\times \text{lo}g_{10}\left(\mathrm{number of interface contacts}\right)$$

## Data Availability

The datasets presented in this study can be found in online repositories. The names of the repository/repositories and accession number(s) can be found in the article/Supplementary Material.
