# Supplementary material for: Forecasting SARS-CoV-2 spike protein evolution from small data by deep learning and regression
Source: Front Syst Biol. 2024 Apr 9;4:1284668. doi: 10.3389/fsysb.2024.1284668 (PMC12341966; doi:10.3389/fsysb.2024.1284668)
Supplement: Supplementary file 2 [file DataSheet4.pdf]

Supplementary Material

Forecasting SARS-CoV-2 spike protein evolution from small data by deep learning and regression

Samuel King[1-3], Xinyi E. Chen[1,4,5], Sarah W. S. Ng[1,4,5], Kimia Rostin[1,4,5], Samuel V. Hahn[1,6], Tylo Roberts[1,4], Janella C. Schwab[1,7], Parneet Sekhon[1,4], Madina Kagieva[1-3], Taylor Reilly[1-3], Ruo Chen Qi[1,8], Paarsa Salman[1-3], Ryan J. Hong[1,4], Eric J. Ma[9], Steven J. Hallam[1,4,10-13]*

[1]International Genetically Engineered Machine (iGEM) Team, University of British Columbia, Vancouver, BC, Canada.
[2]Department of Botany, University of British Columbia, Vancouver, BC, Canada.
[3]Department of Zoology, University of British Columbia, Vancouver, BC, Canada.
[4]Department of Microbiology and Immunology, University of British Columbia, Vancouver, BC, Canada.
[5]Department of Computer Science, University of British Columbia, Vancouver, BC, Canada.
[6]Department of Chemical and Biological Engineering, University of British Columbia, Vancouver, BC, Canada.
[7]Faculty of Land and Food Systems, University of British Columbia, Vancouver, BC, Canada.
[8]Department of Cellular, Anatomical, and Physiological Sciences, University of British Columbia, Vancouver, BC, Canada.
[9]Independent Researcher, Cambridge, MA, USA.
[10]Graduate Program in Bioinformatics, University of British Columbia, Vancouver, BC, Canada.
[11]Genome Science and Technology Program, University of British Columbia, Vancouver, BC, Canada.
[12]Life Sciences Institute, University of British Columbia, Vancouver, BC, Canada.
[13]ECOSCOPE Training Program, University of British Columbia, Vancouver, BC, Canada.

*** Correspondence:**
Steven J. Hallam
shallam@mail.ubc.ca

1 Supplementary Data

Datasets and code associated with this work can be found in the UBC iGEM GitHub repository for VPRE: https://github.com/UBC-iGEM/VPRE.

Supplementary File 1. Spike protein sequences acquired from NCBI GenBank for training the variational autoencoder and Gaussian process algorithms.

Supplementary File 2. Spike protein sequences simulated based on NCBI GenBank sequence set.

Supplementary File 3. Spike protein sequences collected in Australia from January to December 2020.

2 Supplementary Figures and Tables

2.1 Supplementary Figures

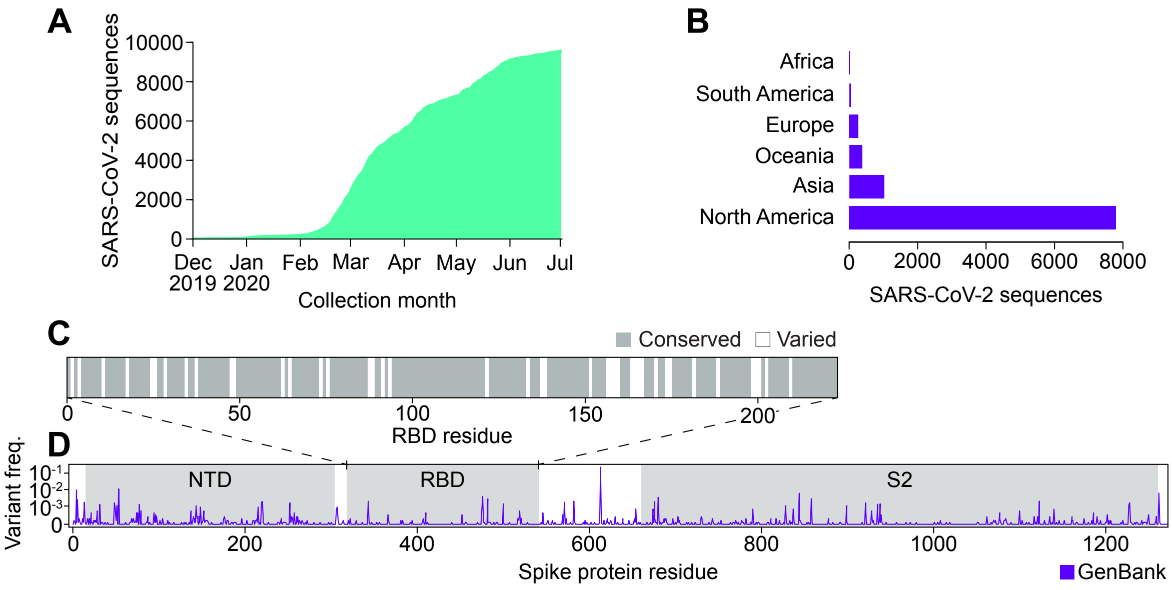

Supplementary Figure 1. Collected sequences for neural network training and encoding. (**A**) Cumulative distribution of sequences over time from the dataset downloaded from NCBI GenBank on August 16th, 2020 (n = 9534). (**B**) Continental distribution of sequences from the GenBank dataset. (**C**) Distribution of amino acid variations in the spike receptor binding domain (white = at least one variant observed from the GenBank dataset; grey = no variant detected in the GenBank dataset). NTD, N-terminal domain; RBD, receptor binding domain; S2, S2 subunit. (**D**) Variant frequency at each amino acid position on spike proteins observed from the NCBI dataset.

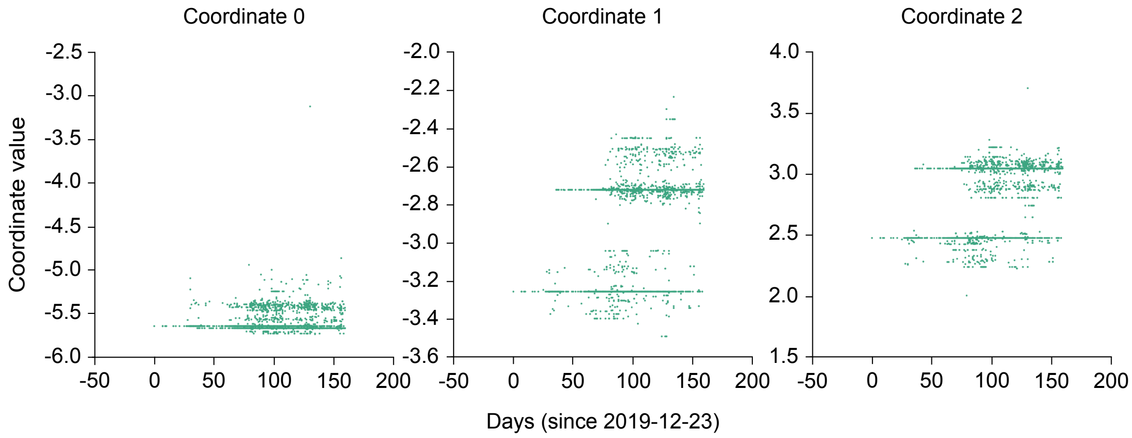

Supplementary Figure 2. Training sequence coordinate encodings across time.

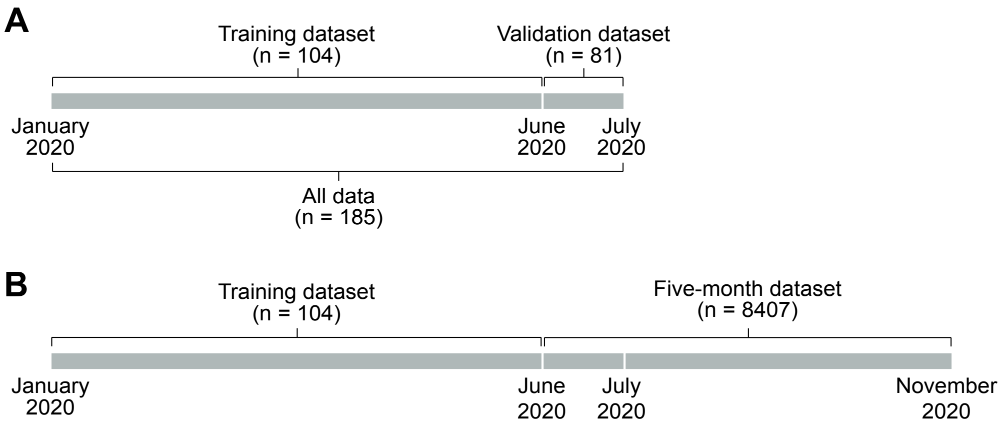

Supplementary Figure 3. Training and validation datasets for the Gaussian process. (**A**) Timeline of the initial training and validation datasets from Australia used for the Gaussian process (GP) regression. (**B**) Timeline of the five-month dataset added onto the prior training dataset used for the GP regression.

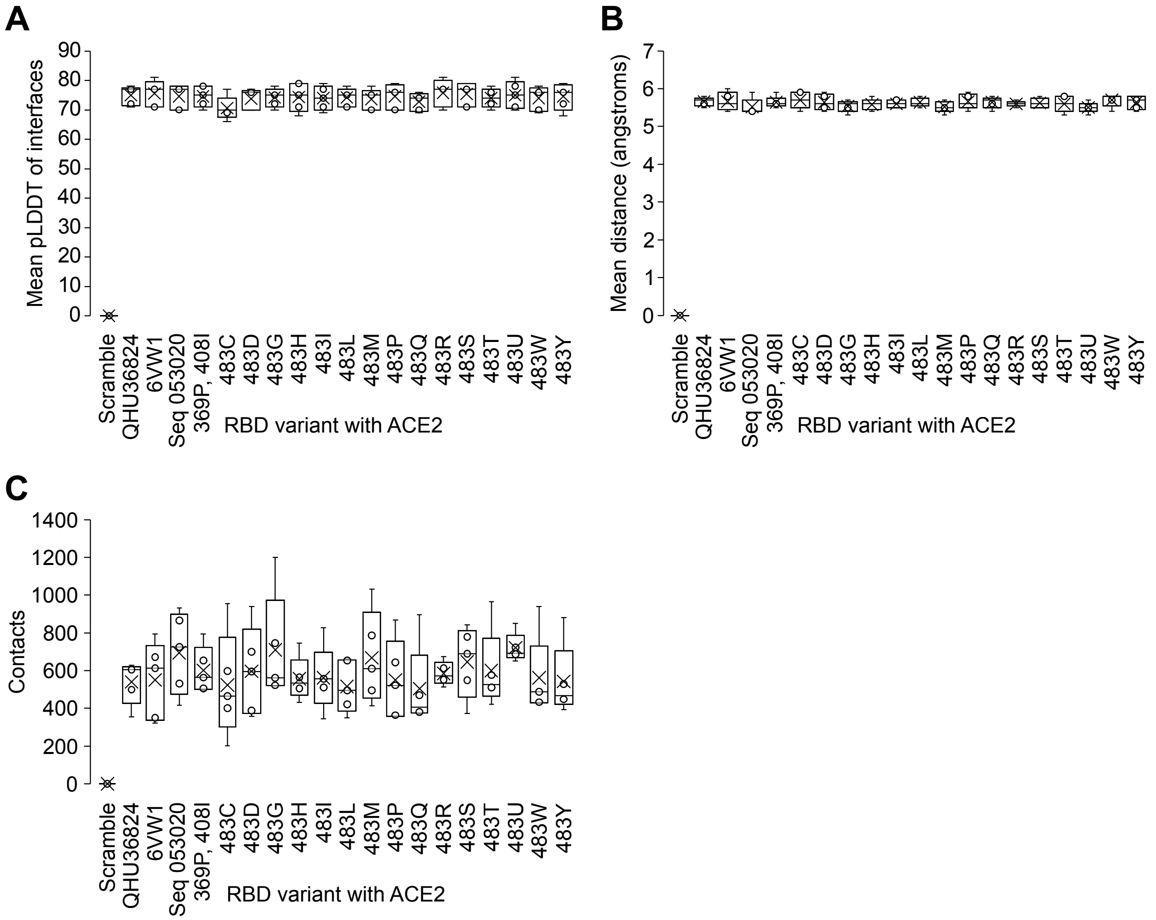

Supplementary Figure 4. Supplementary data for the *in silico* RBD-ACE2 binding analysis. (**A**) Mean pLDDT scores of interfaces in RBD-ACE2 docking models, where a score >90 is considered very high, 80 is confident, 70 is OK, 60 is low, and <50 is very low. (**B**) Mean distances of interfaces. (**C**) Number of contacts between the RBD and ACE2. In each box, x = mean, middle line = median, lower hinge = 25th percentile of the data, upper hinge = 75th percentile of the data, whiskers = 1.5 * interquartile range.

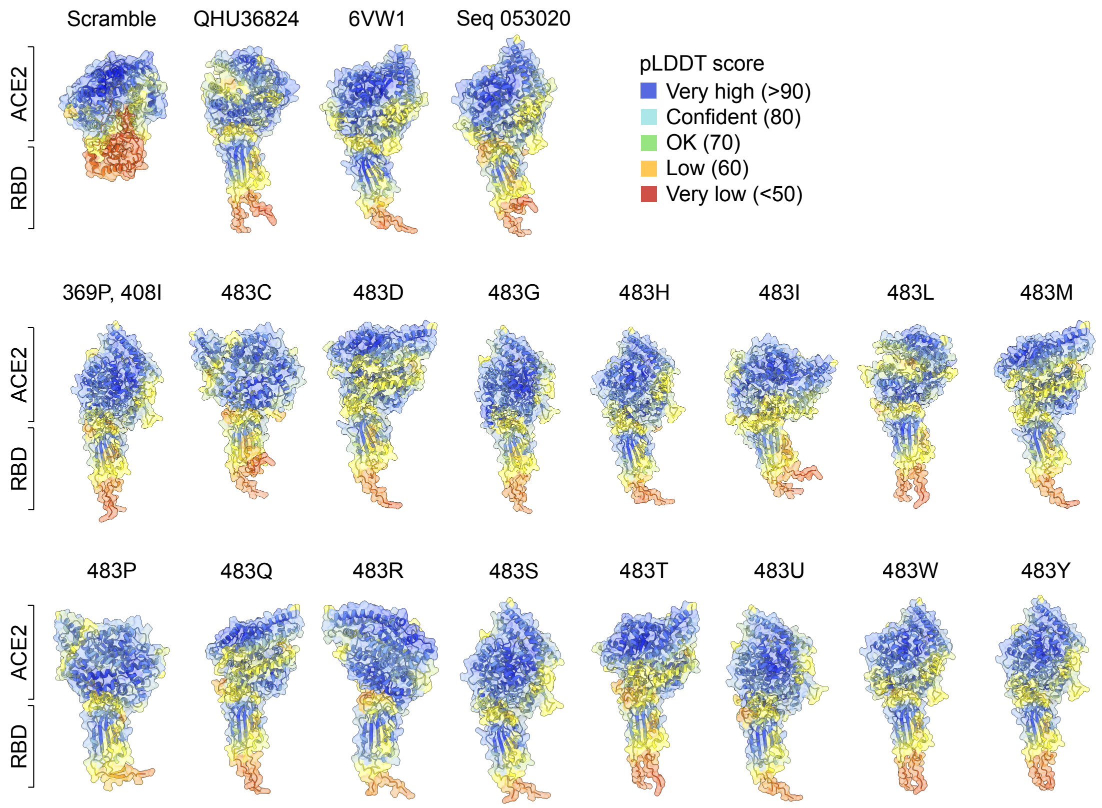

Supplementary Figure 5. AlphaFold2 models of novel RBD-ACE2 complexes colored by pLDDT.

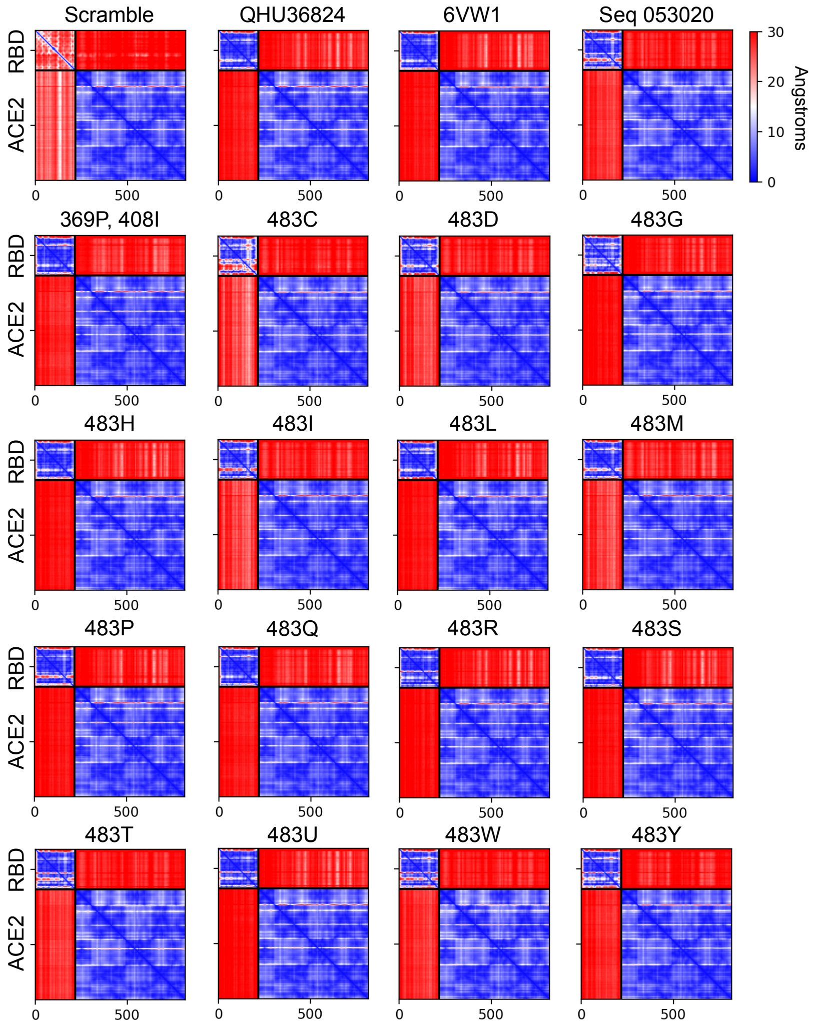

Supplementary Figure 6. Predicted alignment error plots of novel RBD-ACE2 complexes.

2.2 Supplementary Tables

Please refer to the supplementary tables file.
